# The global burden, risk and inequality of maternal obstructed labor and uterine rupture from 1990 to 2019

**DOI:** 10.1186/s12889-024-19429-2

**Published:** 2024-07-29

**Authors:** Mingxing Yan, Hui Li, Xinye Zheng, Feng Li, Chen Gao, Liying Li

**Affiliations:** 1 Department of Obstetrics, Fujian Maternity and Child Health Hospital, College of Clinical Medicine for Obstetrics & Gynecology and Pediatrics, Fujian Medical University, 18 Daoshan Road, Gulou District, Fuzhou, 350001 China; 2 Fujian Clinical Research Center for Maternal-Fetal Medicine, Fuzhou, China; 3 National Key Obstetric Clinical Specialty Construction Institution of China, Fuzhou, China; 4 Clinical Oncology School of Fujian Medical University, Fujian Cancer Hospital, 420 Fuma Road, Jin’an District, Fuzhou, 350014 Fujian China; 5 Department of Obstetrics and gynecology, Ningde Hospital Affiliated to Ningde Normal University, Ningde, China

**Keywords:** Maternal obstructed labor, Uterine rupture, Global burden, Health inequality

## Abstract

**Background:**

Obstructed labor (OL) and uterine rupture (UR) are common obstetric complications. This study explored the burden, risk factors, decomposition, and health inequalities associated with OL and UR to improve global maternal health.

**Methods:**

This was a cross-sectional analysis study including data on OL and UR from the Global Burden of Diseases, and Risk Factors Study (GBD) 2019. The main outcome measures included the number and age-standardized rate (ASR) of incidence, disability-adjusted life years (DALYs), prevalence, and deaths.

**Results:**

The global burden of OL and UR has declined, with a decrease in incidence (number in 2019: 9,410,500.87, 95%UI 11,730,030.94 to 7,564,568.91; ASR in 2019: 119.64 per 100,000, 95%UI 149.15 to 96.21; estimated annual percentage change [EAPC] from 1990 to 2019: -1.34, 95% CI -1.41 to -1.27) and prevalence over time. However, DALYs (number in 2019: 999,540.67, 95%UI 1,209,749.35 to 817,352.49; ASR in 2019: 12.92, 95%UI 15.63 to 10.56; EAPC from 1990 to 2019: -0.91, 95% CI -1.26 to -0.57) and deaths remain significant. ASR of DALYs increased for the 10–14 year-old age group (2.01, 95% CI 1.53 to 2.5), the 15–19 year-old age group (0.07, 95% CI -0.47 to 0.61), Andean Latin America (3.47, 95% CI 3.05 to 3.89), and Caribbean (4.16, 95% CI 6 to 4.76). Iron deficiency was identified as a risk factor for OL and UR, and its impact varied across different socio-demographic indices (SDIs). Decomposition analysis showed that population growth primarily contributed to the burden, especially in low SDI regions. Health inequalities were evident, the slope and intercept for DALYs were − 47.95 (95% CI -52.87 to -43.02) and − 29.29 (95% CI -32.95 to -25.63) in 1990, 39.37 (95%CI 36.29 to 42.45) and 24.87 (95%CI 22.56 to 27.18) in 2019. Concentration indices of ASR-DALYs were − 0.2908 in 1990 and − 0.2922 in 2019.

**Conclusion:**

This study highlights the significant burden of OL and UR and emphasizes the need for continuous efforts to reduce maternal mortality and morbidity. Understanding risk factors and addressing health inequalities are crucial for the development of effective interventions and policies to improve maternal health outcomes globally.

**Supplementary Information:**

The online version contains supplementary material available at 10.1186/s12889-024-19429-2.

## Background

Obstructed labor (OL) and uterine rupture (UR) are common obstetric complications that pose a significant threat to the health of mothers and infants [1]. In recent years, there has been increasing recognition of the need to address maternal health and reduce maternal mortality and morbidity [2]. OL and UR are major contributors to adverse maternal outcomes, leading to complications such as infection, hysterectomy, and maternal death [3, 4]. These conditions often result from a lack of access to quality obstetric care, inadequate healthcare infrastructure, and socio-economic disparities [1, 5]. Pregnant women facing these challenges, particularly those in low- and middle-income countries, are more susceptible to experiencing OL and UR [6].

Despite existing studies reporting the burden of these conditions globally, comprehensive research on the burden, risk factors, and health inequalities among different regions and populations is lacking [7]. Therefore, it is essential to delve into these issues to enhance understanding of the epidemiological characteristics of OL and UR and to provide a scientific basis for developing effective prevention and intervention measures.

Moreover, there is a growing recognition of the impact of health inequalities on maternal health outcomes [1, 2]. Disparities in access to care, socioeconomic status, and education can contribute to inequitable distribution and management of obstetric emergencies such as OL and UR [8]. Understanding the burden of OL and UR, as well as their associated risk factors and health inequalities, can inform policymakers, healthcare professionals, and researchers in the development of targeted interventions and strategies [9]. Furthermore, understanding these related underlying factors can help to advocate for improved access to quality obstetric care and addressing the underlying social determinants of health that contribute to adverse maternal outcomes [10].

Thus, this study aimed to explore the burdens, risk factors, decomposition, and health inequalities associated with OL and UR. By providing a comprehensive analysis of these aspects, the results aim to contribute to the existing evidence base and facilitate the development of evidence-based policies and interventions to improve global maternal health outcomes.

## Materials and methods

### Data source

The study utilized data from the Global Burden of Diseases, and Risk Factors Study (GBD) 2019. The GBD provides comprehensive and standardized data on disease burden, risk factors, and mortality from various sources, including health surveys, vital registration systems, and published literature from 21 regions and 195 countries worldwide.

### Study design

We used a cross-sectional approach to analyze the burden, risk factors, decomposition, and health inequalities associated with OL and UR. The outcomes measured were number and age-standardized rates of incidence, disability-adjusted life years (DALYs), prevalence, and deaths.

### Statistical analyses

#### Burden and risk factor analysis

An initial comprehensive analysis of the burden and risk factors associated with OL and UR was performed. We compiled the numbers and age-standardized rates (ASR; per 100,000 individuals) of incident, DALYs, prevalence, and deaths at the global, age-group, Socio-Demographic Index (SDI), and GBD regions levels for the 2019. Then, trend changes from 1990 to 2019, the estimated annual percentage change (EAPC) for ASR of incident cases, DALYs, prevalence, and deaths were calculated to understand the tempo of change over the study period.

$$\eqalign{{\rm{EAPC = }} & {\rm{100 \times (exp(\beta )}}\,{\rm{ - }}\,{\rm{1),\,y = \alpha }}\,{\rm{ + }}\,{\rm{\beta x}}\,{\rm{ + }}\,{\rm{\varepsilon ,}} \cr {\rm{ }} & {\rm{y = In}}\left( {{\rm{ASR}}} \right){\rm\,{, x = year}}\cr} $$

To facilitate the interpretation of these indicators, we constructed bivariate plots to visualize the trends in the number and rate of incidents, DALYs, prevalence, and deaths from 1990 to 2019, including their 95% confidence intervals (95%CIs). Subsequently, line graphs were plotted to display the trends disaggregated by age group, for the same period. The distribution of disease burden was then mapped across all countries and regions. We superimposed the ASR- and number-based incidence, DALYs, prevalence, and deaths data for the years 1990 and 2019 onto world maps to provide a spatial representation of the disease burden. Only one risk factor was identified in the GBD; we also presented time-series data on the rates of DALYs and deaths from 1990 to 2019 to illustrate the temporal trends associated with GBD.

#### Inequality analysis

A decomposition analysis was conducted to understand the contribution of population base, aging, and epidemiological change to the disease burden across different levels of SDI to identify the primary factors influencing the disease burden at various levels of development.

To further investigate health inequalities further, we computed the slope index and concentration indices for the incidence, DALYs, prevalence, and deaths from 1990 to 2019. These indices provide insight into the inequality in the disease’s burden over time, with a focus on how uneven regional development may have affected health outcomes. Linear regression models and heteroscedasticity were used to calculate and test skewness indices. Heteroscedasticity was weighted using repeated iterations of robust regression to obtain the slope and intercept of the slope index. In addition, we used Health Equity Assessment Toolkit (HEAT) Plus calculations containing difference, ratio, absolute concentration index (ACI), relative concentration index (RCI), population attributable fraction (PAF), and population attributable risk (PAR).

#### Statistical software

Concentration indices were calculated using the HEAT Plus software from the World Health Organization (version 5.0). All formulas and their explanations are available in the HEAT Plus Technical Notes (available at https://www.who.int/data/inequality-monitor/assessment_toolkit). All remaining statistical analyses were performed using R software (version 4.3.2). Adobe Illustration 2023 was used for all image enhancement. Statistical significance was set at *P* < 0.05.

## Results

### Disease burden and trends at the global, age, SDI, GBD region, and country levels

The numbers and ASR of incident cases, DALYs, prevalence, and deaths due to OL and UR in 2019 are presented in Table 1 and Supplemental Table 1, along with the EAPC from 1990 to 2019. In 2019, there were an estimated 9,410,500.87 (95%UI 11,730,030.94–7,564,568.91) incident cases of the disease, with an ASR of 119.64 per 100,000 (95%UI 149.15–96.21). The number of DALYs exceeded 10% of the incidence, with 999,540.67 (95%UI 1,209,749.35–817,352.49) DALYs and an ASR of 12.92 (95%UI 15.63–10.56). From 1990 to 2019, the ASR-incidence decreased significantly, with an EAPC of -1.34 (95% CI -1.41 to -1.27), which was more than ASR-DALYs (-0.91, 95% CI -1.26 to -0.57). Among the GBD regions, High-income Asia Pacific (0.19, 95% CI -0.1 to 0.48), Australasia (0.29, 95% CI 0.16 to 0.42), Eastern Europe (0.45, 95% CI -0.09 to 0.99), and Western Europe (0.37, 95% CI 0.26 to 0.49) experienced an increase in ASR of incident cases. ASR of DALYs increased for the 10–14-year-old age group (2.01, 95% CI 1.53 to 2.5) and the 15–19-year-old age group (0.07, 95% CI -0.47 to 0.61). Furthermore, ASR of DALYs increased in Andean Latin America (3.47, 95% CI 3.05 to 3.89), Caribbean (4.16, 95% CI 6 to 4.76), Australasia (0.24, 95% CI 0.09 to 0.38), Oceania (0.23, 95%CI -0.05 to 0.5), and Western Europe (0.11, 95% CI -0.01 to 0.22) regions.

**Table 1:** The number and ASR of incident and DALYs for maternal obstructed labor and uterine rupture in 2019 and changing trends from 1990 to 2019

	2019		1990–2019	2019		1990–2019
	Incidence cases (95% UI)	ASIR per 100,000 (95% UI)	EAPC of ASIR (95% CI)	DALYs cases (95% UI)	ASR-DALYs per 100,000 (95% UI)	EAPC of ASR-DALYs (95% CI)
Global	9,410,500.87 (11,730,030.94–7,564,568.91)	119.64 (149.15–96.21)	-1.34 (-1.41 to -1.27)	999,540.67 (1,209,749.35–817,352.49)	12.92 (15.63–10.56)	-0.91 (-1.26 to -0.57)
Age (years)						
0–9 years	0.00 (0.00–0.00)	0.00 (0.00–0.00)	0.00 (0.00–0.00)	0.00 (0.00–0.00)	0.00 (0.00–0.00)	0.00 (0.00–0.00)
10–14 years	24,825.43 (34,865.65–16,641.62)	3.87 (5.43–2.59)	-0.48 (-0.61 to -0.35)	2,848.48 (3,963.07–2,069.96)	0.44 (0.62 − 0.32)	2.01 (1.53 to 2.5)
15–19 years	976,821.90 (1,297,639.82–704,780.38)	157.67 (209.45–113.76)	-2.04 (-2.1 to -1.97)	73,508.50 (93,260.32–57,595.31)	11.86 (15.05–9.30)	0.07 (-0.47 to 0.61)
20–24 years	2,339,763.53 (3,265,760.58–704,780.38)	389.87 (544.16–272.92)	-1.9 (-1.95 to -1.86)	168,528.86 (213,097.14–132,034.60)	28.08 (35.51–22.00)	-1 (-1.47 to -0.53)
25–29 years	2,584,846.60 (3,555,523.69–1,822,346.45)	426.92 (587.23–300.98)	-1.31 (-1.39 to -1.24)	192,536.71 (244,326.34–149,941.28)	31.08 (40.35–24.76)	-1.19 (-1.5 to -0.87)
30–34 years	2,004,631.36 (2,726,772.08–1,450,516.93)	333.14 (453.15–241.06)	-0.62 (-0.73 to -0.51)	202,044.42 (267,809.01–155,213.87)	33.58 (44.51–25.79)	-1.03 (-1.38 to -0.68)
35–39 years	1,052,867.51 (1,453,159.25–748,489.90)	194.62 (268.62–138.36)	-0.41 (-0.57 to -0.25)	166,611.18 (217,323.28–130,968.35)	30.80 (40.17–24.21)	-0.65 (-1.05 to -0.25)
40–44 years	339,736.68 (464,687.40–242,377.08)	68.85 (94.17–49.12)	-0.9 (-1.14 to -0.66)	106,370.75 (135,756.64–84,861.35)	21.56 (27.51–17.20)	-0.65 (-0.97 to -0.32)
45–49 years	84,975.61 (121,716.99–57,055.48)	17.93 (25.69–12.04)	-1.67 (-2.05 to -1.3)	55,562.01 (70,691.20–42,764.27)	11.73 (14.92–9.03)	-0.75 (-0.95 to -0.55)
50–54 years	2,032.24 (2,645.73–1,536.48)	0.47 (0.61 − 0.35)	-1.8 (-2.12 to -1.49)	14,336.48 (21,237.49–9,274.25)	3.28 (4.86–2.12)	-2.19 (-2.4 to -1.99)
55 + years	0.00 (0.00–0.00)	0.00 (0.00–0.00)	0.00 (0.00–0.00)	0.00 (0.00–0.00)	0.00 (0.00–0.00)	0.00 (0.00–0.00)
SDI						
High	1,221,481.07 (1,546,427.56–953,898.30)	130.52 (163.85–102.27)	-1.51 (-1.75 to -1.27)	6,327.62 (9,600.99–3,701.73)	0.62 (0.95 − 0.37)	-1.39 (-1.59 to -1.18)
High-middle	1,360,919.16 (1,688,288.37–1,077,881.33)	95.42 (118.26–75.92)	-0.83 (-1.04 to -0.63)	26,391.90 (33,273.48–20,736.82)	1.85 (2.33–1.45)	-2.78 (-3.02 to -2.54)
Middle	2,330,998.59 (2,980,660.75–1,826,717.65)	93.37 (119.36–73.11)	-1.44 (-1.62 to -1.27)	142,807.10 (175,079.70–115,852.64)	5.96 (7.31–4.83)	-1.79 (-1.96 to -1.62)
Low-middle	2,373,240.05 (3,016,707.12–1,872,108.34)	122.20 (155.04–96.92)	-2.18 (-2.21 to -2.16)	389,052.84 (479,855.18–318,673.90)	22.06 (27.20–18.07)	-1.98 (-2.44 to -1.52)
Low	2,118,116.85 (2,619,399.14–1,734,916.22)	183.29 (224.60–150.08)	-1.49 (-1.53 to -1.45)	434,446.86 (535,019.16–350,446.86)	38.49 (47.40–31.05)	-1.22 (-1.47 to -0.97)
GBD region						
Central Asia	130,119.06 (173,231.80–97,223.44)	127.93 (171.21–96.19)	-0.71 (-1.03 to -0.4)	963.59 (1,375.20–631.18)	1.03 (1.47–0.67)	-1.13 (-1.5 to -0.76)
East Asia	806,830.44 (1,036,003.23–636,511.85)	54.56 (70.13–42.57)	-1.74 (-2.12 to -1.36)	11,127.79 (14,244.22–8,415.67)	0.76 (0.97 − 0.57)	-4.17 (-4.74 to -3.59)
South Asia	2,530,142.29 (3,298,887.34–1,955,819.36)	124.56 (162.35–96.57)	-2.39 (-2.43 to -2.35)	378,270.86 (483,470.57–290,365.16)	20.95 (26.78–16.08)	-3.43 (-3.96 to -2.9)
Southeast Asia	667,369.43 (891,416.76–501,770.96)	92.40 (123.67–69.43)	-1.55 (-1.6 to -1.49)	13,798.39 (16,955.43–11,157.29)	2.05 (2.52–1.66)	-1.48 (-1.84 to -1.11)
High-income Asia Pacific	104,387.83 (137,387.10–78,575.55)	67.30 (88.36–50.54)	0.19 (-0.1 to 0.48)	547.80 (851.69–311.48)	0.29 (0.45 − 0.17)	-1.53 (-1.71 to -1.34)
North Africa and Middle East	1,070,648.56 (1,398,164.13–823,364.80)	158.14 (206.95–121.31)	-2.08 (-2.18 to -1.99)	62,092.96 (78,355.86–48,793.46)	10.20 (12.87–8.02)	-1.92 (-1.96 to -1.89)
Central Sub-Saharan Africa	158,925.50 (197,755.20–123,608.78)	121.26 (150.68–95.06)	-1.35 (-1.52 to -1.18)	20,946.10 (27,524.50–15,613.95)	15.92 (20.92–11.87)	-1.27 (-1.42 to -1.11)
Eastern Sub-Saharan Africa	772,939.62 (937,611.47–632,168.25)	181.29 (218.64–148.59)	-1.28 (-1.32 to -1.24)	240,996.26 (303,397.10–190,069.14)	58.53 (73.68–46.16)	-0.45 (-0.68 to -0.21)
Southern Sub-Saharan Africa	67,059.13 (82,427.62–54,164.45)	75.73 (93.20–61.28)	-1.22 (-1.26 to -1.19)	12,575.88 (17,407.16–8,888.67)	16.00 (22.15–11.31)	-1.31 (-1.74 to -0.88)
Western Sub-Saharan Africa	699,427.82 (861,391.19–559,807.72)	154.61 (187.98–124.62)	-1.11 (-1.42 to -0.8)	232,515.59 (297,270.27–181,454.96)	50.96 (65.15–39.77)	-0.13 (-0.28 to 0.01)
Andean Latin America	130,340.48 (164,424.79–103,266.35)	192.64 (243.02–152.79)	-1.59 (-1.7 to -1.49)	1,896.01 (2,470.93–1,388.44)	2.98 (3.89–2.18)	3.47 (3.05 to 3.89)
Central Latin America	309,063.74 (379,044.24–250,095.33)	116.07 (142.04–94.22)	-2.05 (-2.25 to -1.85)	6,971.23 (8,716.99–5,544.34)	2.79 (3.49–2.22)	-1.16 (-1.36 to -0.96)
Southern Latin America	240,630.57 (273,535.61–195,307.61)	355.06 (403.92–287.89)	-0.92 (-1.02 to -0.81)	12,575.88 (17,407.16–8,888.67)	1.88 (2.92–1.07)	-0.14 (-0.31 to 0.03)
Tropical Latin America	92,309.67 (113,960.46–74,598.73)	39.94 (49.28–32.28)	-1.21 (-1.39 to -1.03)	2,656.98 (3,337.05–2,041.75)	1.19 (1.49–0.91)	-2.27 (-2.57 to -1.97)
High-income North America	485,910.16 (597,347.47–395,078.34)	146.72 (180.26–119.04)	-2.99 (-3.54 to -2.44)	2,501.43 (3,785.01–1,471.08)	0.69 (1.04–0.40)	-2.09 (-2.57 to -1.6)
Caribbean	39,936.54 (51,815.30–30,651.24)	83.49 (108.19–64.08)	-1.04 (-1.1 to -0.97)	849.21 (1,073.88–663.66)	1.80 (2.28–1.41)	4.16 (3.56 to 4.76)
Australasia	73,258.76 (92,478.72–55,572.49)	267.40 (336.85–203.94)	0.29 (0.16 to 0.42)	308.93 (539.77–136.93)	1.06 (1.86 − 0.47)	0.24 (0.09 to 0.38)
Oceania	24,856.08 (33,004.29–18,759.94)	177.73 (235.30–134.32)	-0.43 (-0.45 to -0.41)	4,356.09 (6,240.42–2,960.86)	32.81 (47.00–22.30)	0.23 (-0.05 to 0.5)
Central Europe	42,475.52 (56,466.85–31,781.61)	42.54 (55.87–31.83)	-0.56 (-0.94 to -0.18)	274.82 (397.26–177.22)	0.24 (0.35 − 0.16)	-0.76 (-1.04 to -0.48)
Eastern Europe	265,696.39 (350,808.71–199,614.48)	145.34 (192.96–108.25)	0.45 (-0.09 to 0.99)	1,584.58 (2,390.05–960.83)	0.75 (1.14–0.46)	-1.56 (-1.71 to -1.41)
Western Europe	698,173.27 (888,169.07–532,696.94)	187.54 (238.08–143.50)	0.37 (0.26 to 0.49)	3,049.77 (4,922.75–1,589.10)	0.70 (1.13–0.36)	0.11 (-0.01 to 0.22)

ASR, age-standardized rate; DALYs, disability-adjusted life years; EAPC, estimated annual percentage change; UI, uncertainty interval; CI, confidence interval; SDI, Sociodemographic Index; GBD, global burden of disease

**Fig. 1:**
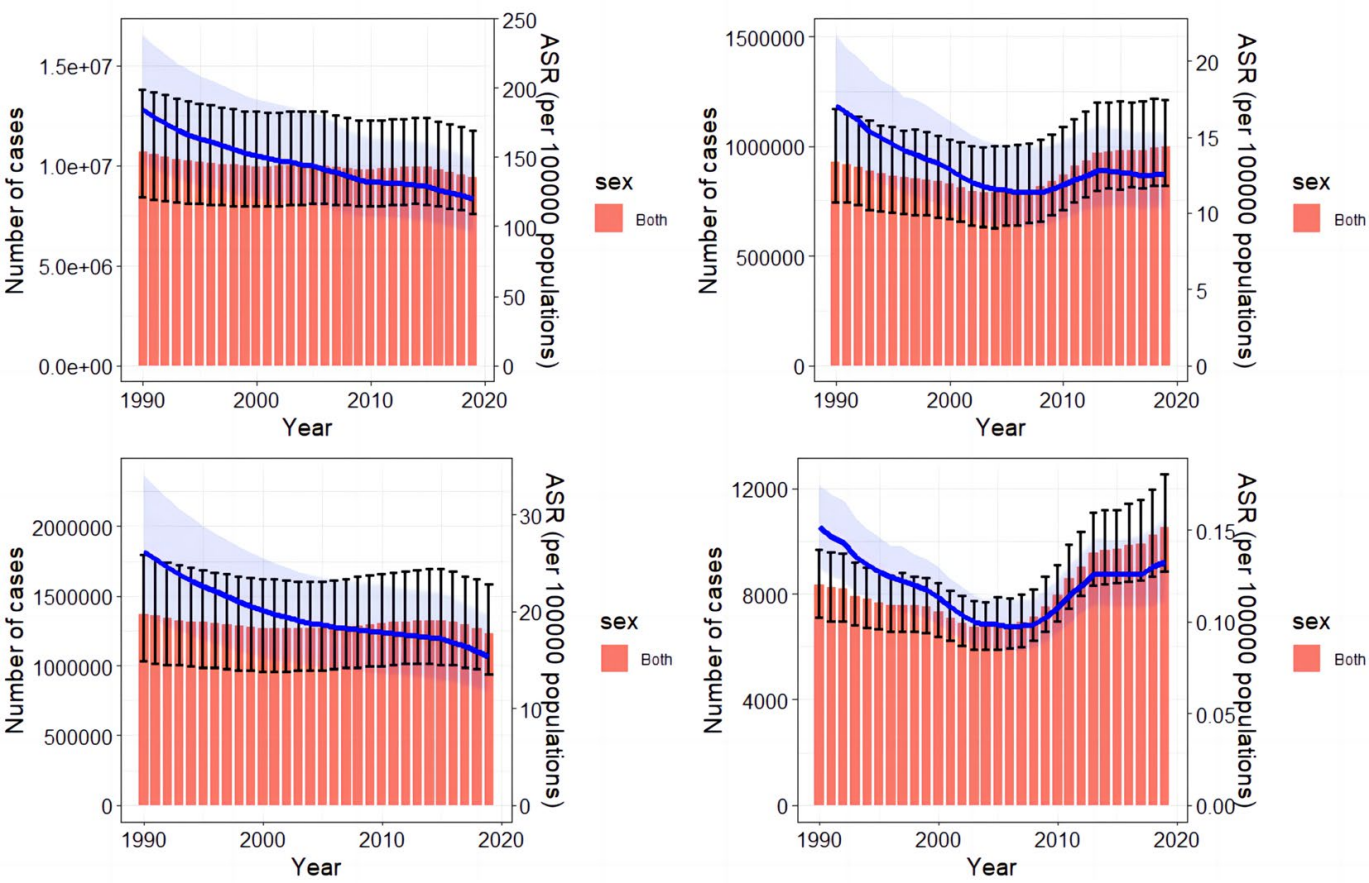
Bar and line charts for global burden of maternal obstructed labor and uterine rupture from 1990 to 2019. (**A**) Bar chart of the number-incidence and line chart of ASR-incidence; (**B**) Bar chart of the number-DALYs and line chart of ASR-DALYs; (**C**) Bar chart of the number-prevalence and line chart of ASR-prevalence; (**D**) Bar chart of the number-deaths and line chart of ASR-deaths. ASR, age-standardized rates; DALYs, disability-adjusted life years

**Fig. 2:**
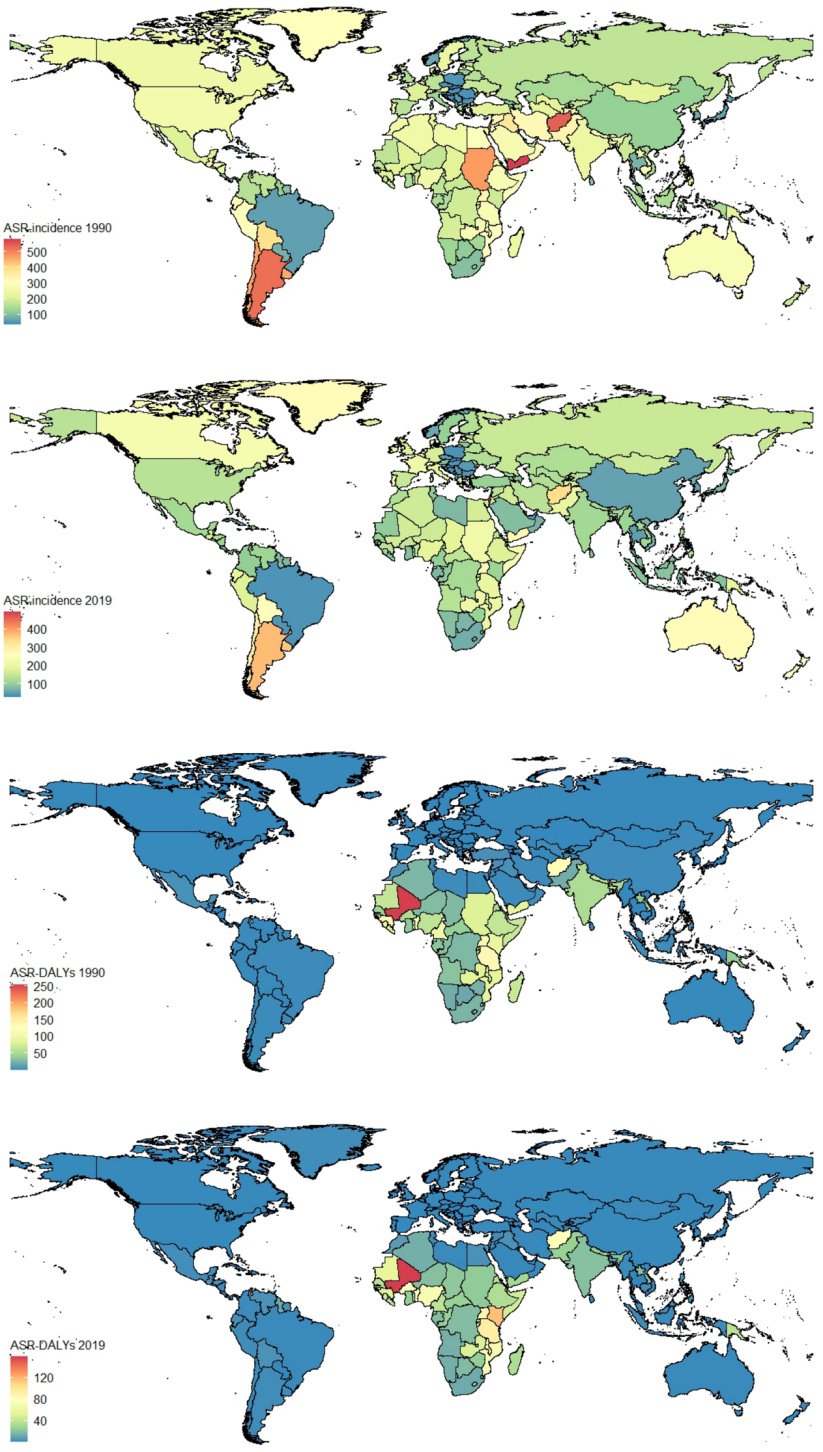
Global distribution maps for the burden of maternal obstructed labor and uterine rupture in 204 countries and territories in 1990 and 2019. (**A**) ASR-incidence in 1990; (**B**) ASR-incidence in 2019; (**C**) ASR-DALYs in 1990; (**D**) ASR-DALYs in 2019. ASR, age-standardized rates; DALYs, disability-adjusted life years

The changes in incidence, DALYs, prevalence, and deaths are shown in Fig. 1, indicating a continuous decrease in incidence and prevalence on a global scale (Fig. 1A and C). DALYs and deaths declined until 2008, followed by gradual increases. The trends in different age groups from 1990 to 2019 are depicted in Supplemental Fig. S1-S4, revealing that the incidence is highest among the 20–29-year-old age group, DALYs are highest among the 25–34-year-old age group, prevalence is highest among the 30–34-year-old age group, and deaths are highest among the 20–39-year-old age group. Global distribution maps of ASR-incidence and DALYs for OL and UR in 1990 and 2019 are presented in Fig. 2, while ASR-prevalence and deaths are shown in Supplemental Fig. 5, and number-incidence and DALYs are displayed in Supplemental Fig. 6, and number-prevalence and deaths are presented in Supplemental Fig. 7.

### Burden and trends of risk factors

**Fig. 3:**
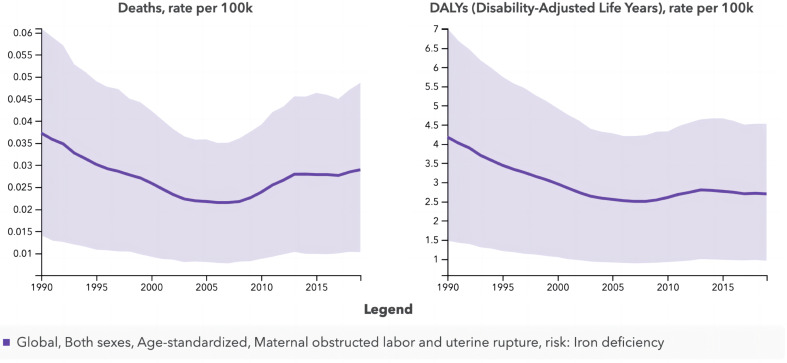
The changing trends in DALYs and deaths associated with iron deficiency globally and across different SDI regions from 1990 to 2019. (**A**) globally ASR- deaths; (**B**) globally ASR- DALYs; (**C**) ASR- deaths by SDI quintile; (**D**) ASR- DALYs by SDI quintile. DALYs, disability-adjusted life years; ASR, age-standardized rates; SDI, Sociodemographic Index

In the GBD, only one risk factor, iron deficiency, was identified for OL and UR. Figure 3 illustrates the changing trends in DALYs and deaths associated with iron deficiency globally and across different SDI regions from 1990 to 2019. The highest risk attributable to iron deficiency was observed in 1990, which decreased until 2005–2010 before gradually increasing thereafter (Fig. 3A-B). Additionally, the higher the SDI, the lower the harm caused by iron deficiency. The ASR-death attributable to iron deficiency in Low SDI regions decreased from 0.14 in 1990 to 0.10 in 2019 approximately, which was close to twice the value in Low-middle SDI regions. The ASR-DALYs in Low SDI regions showed a decrease from 17 in 1990 to 10 in 2019, while in Low-middle SDI regions, it nearly halved from 10 (Fig. 3C-D).

### Decomposition analysis

**Fig. 4:**
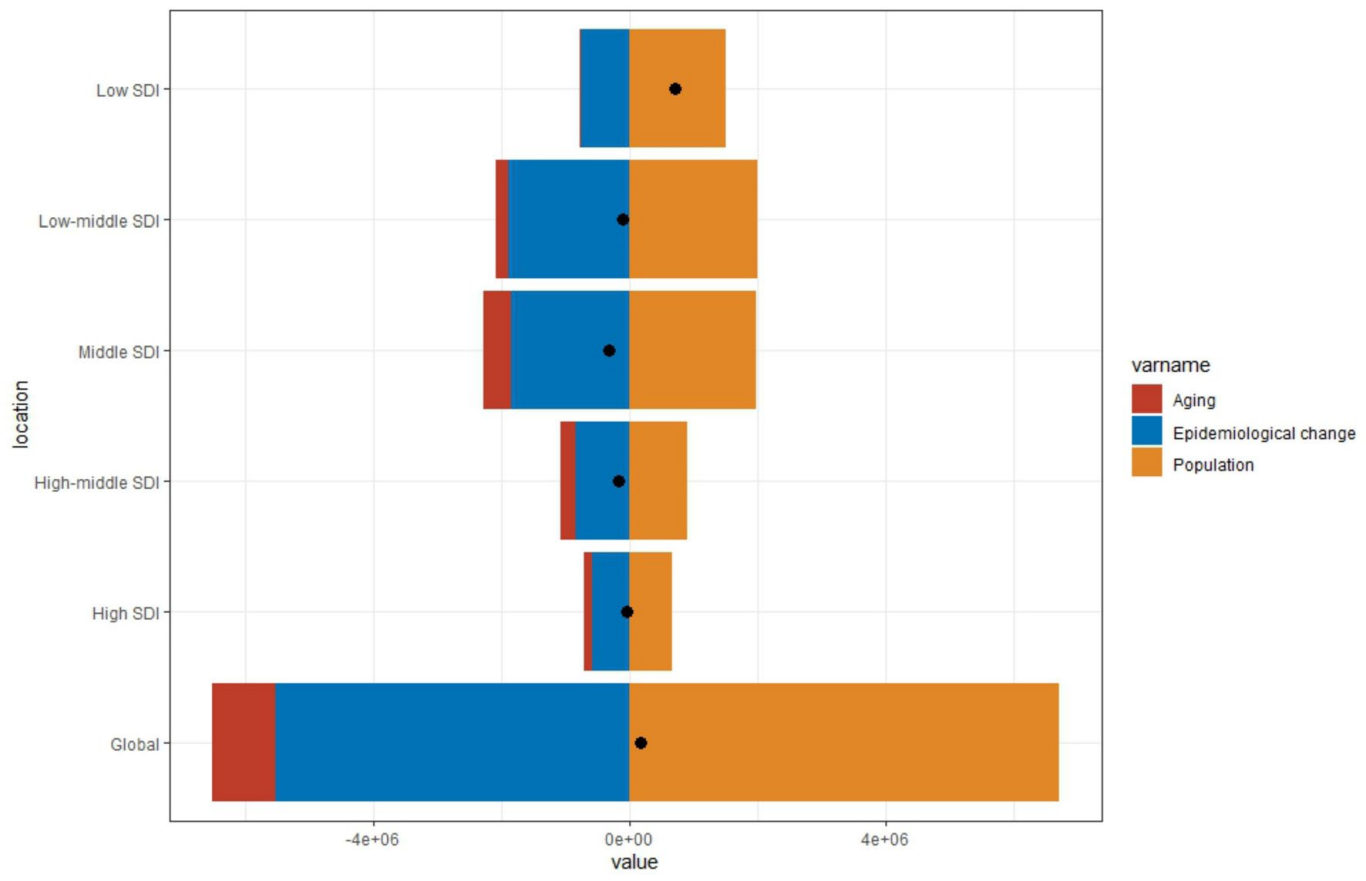
Changes in maternal obstructed labor and uterine rupture incidences rate according to population-level determinants of population growth, aging, and epidemiological change from 1990 to 2019 at the global Level and by SDI quintile. (The black dot represents the overall value of change contributed by all 3 components.) SDI, Sociodemographic Index

To better understand the relative contributions of population base, aging, and the epidemiological trends of OL and UR globally and in different SDI regions, we conducted a decomposition analysis. Incidence (Fig. 4), DALYs (Supplemental Fig. 8), prevalence (Supplemental Fig. 9), and deaths (Supplemental Fig. 10) were decomposed into these factors, respectively. The results showed that the global and low SDI regions were primarily affected by the growth in population base, whereas the others were influenced by epidemiological changes. Middle SDI regions exhibited the largest proportion of aging as a factor. Similar patterns were observed for DALYs (Supplemental Fig. 8). For prevalence, the main factors affecting global and low-middle SDI regions were epidemiological changes, while for low and middle SDI regions, it was population base (Supplemental Fig. 9). Finally, in terms of deaths, global, low, and low-middle SDI regions were greatly influenced by population base growth (Supplemental Fig. 10).

### Slope indices

**Fig. 5:**
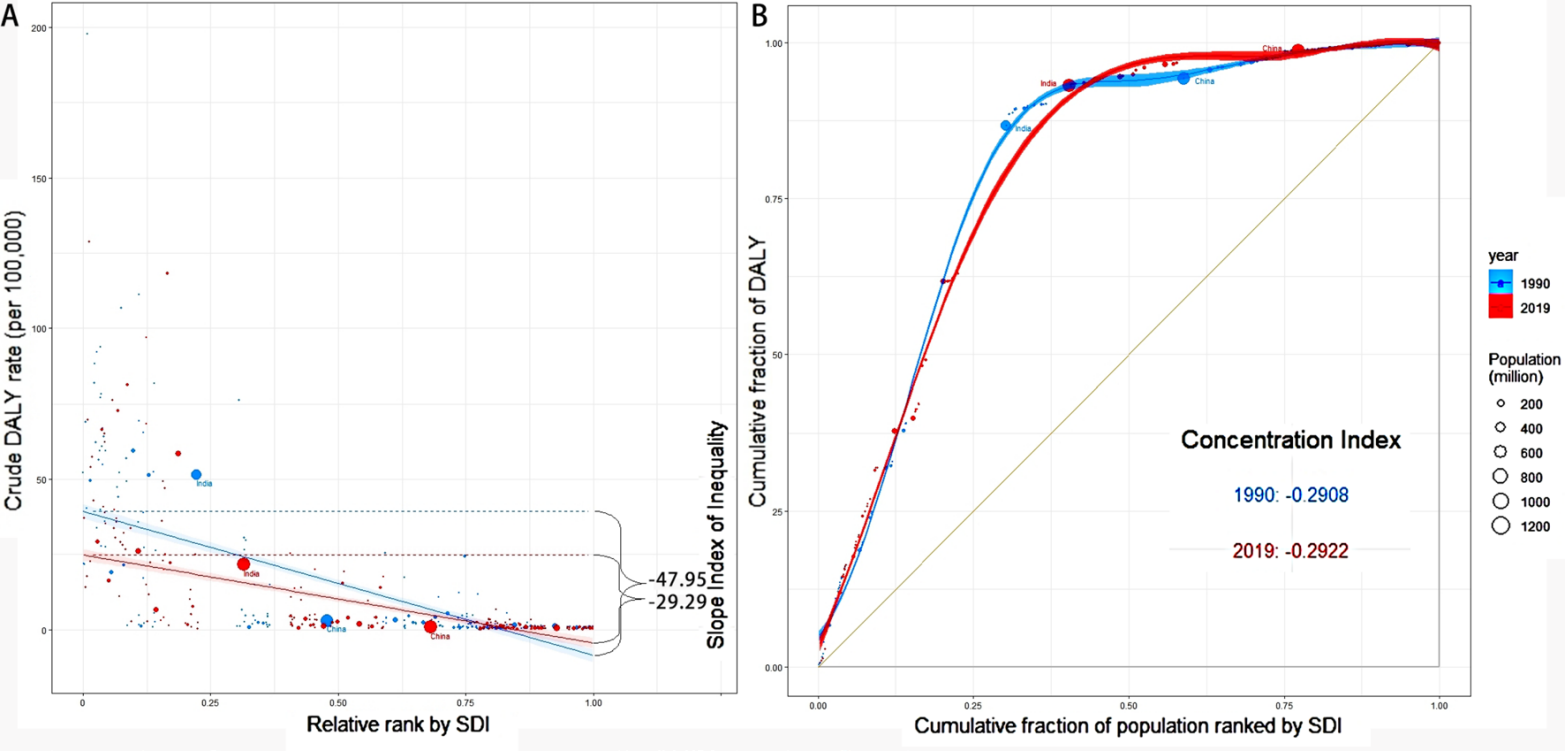
The health inequality indices for ASR-DALYs of maternal obstructed labor and uterine rupture in 1990 and 2019 based on the SDI of 204 countries and territories globally. (**A**) Slope indices (the numbers adjacent to the brackets indicate the slopes); (**B**) Concentration indices. Each country or region is represented by a solid dot, with larger dots indicating a higher population. China and India are highlighted for comparative purposes. SDI, Sociodemographic Index; ASR, age-standardized rates; DALYs, disability-adjusted life years

The slope indices of inequality for ASR of OL and UR in 1990 and 2019 are presented in Table 2. Negative slope values indicate a gradual reduction in disease burden with increasing SDI. The slope and intercept for DALYs (Fig. 5A) in 1990 and 2019 were − 47.95 (95% CI -52.87 to -43.02), -29.29 (95% CI -32.95 to -25.63), 39.37 (95%CI 36.29 to 42.45), and 24.87 (95%CI 22.56 to 27.18), respectively. The slopes of Incidence, Prevalence and deaths all gradually decreased from 1990 to 2019 (Table 2), from − 86.33 (95%CI -123.87 to -48.80) to -45.53 (95%CI -60.85 to -30.22), -60.95 (95%CI − 68.10 to -53.79) to -15.97 (95%CI -18.58 to -13.36), -0.26 (95%CI -0.29 to -0.23) to -0.17 (95%CI -0.19 to -0.15), respectively.

**Table 2:** Slope indices inequality for ASR of maternal obstructed labor and uterine rupture in 1990 and 2019

	Chi-square	*p*-value	Slope (95% CI)	Intercept (95% CI)
Incidence				
1990	2.0767	0.1496	-86.33 (-123.87 to -48.80)	242.30 (218.81 to 265.80)
2019	10.9801	0.0009	-45.53 (-60.85 to -30.22)	152.60 (138.84 to 166.37)
DALYs				
1990	145.98	< 0.0001	-47.95 (-52.87 to -43.02)	39.37 (36.29 to 42.45)
2019	131.75	< 0.0001	-29.29 (-32.95 to -25.63)	24.87 (22.56 to 27.18)
Prevalence				
1990	152.52	< 0.0001	-60.95 (-68.10 to -53.79)	50.13 (45.65 to 54.61)
2019	135.93	< 0.0001	-15.97 (-18.58 to -13.36)	19.88 (17.53 to 22.23)
Deaths				
1990	191.45	< 0.0001	-0.26 (-0.29 to -0.23)	0.22 (0.20 to 0.24)
2019	157.52	< 0.0001	-0.17 (-0.19 to -0.15)	0.21 (0.19 to 0.23)

ASR, age-standardized rate; DALYs, disability-adjusted life years; CI, confidence interval

### Concentration indices and other measures of health inequality

Simple and complex measures of health inequality are shown in Table 3 and Supplemental Tables 2–4, respectively. ASR-DALYs (Table 3; Fig. 5B) of OL and UR with concentration indices, ACI and RCI changed from − 0.2908, -10.25 (95% CI -11.68 to -8.82) and − 59.15 (95% CI -67.60 to -50.71) in 1990, to -0.2922, -7.68 (95% CI -8.39 to -6.97) and − 59.43 (95% CI − 65.75 to -53.10) in 2019. Its PAF and PAR were − 92.98 and − 16.11, -92.28 and − 11.93 in 1990 and 2019, respectively. The ACI (-17.25, -14.51, -15.33, and − 13.22) and RCI (-8.62, -8.97, -10.93, and − 10.86) of ASR-incidence (Supplemental Table 2) were stable in 1990, 2000, 2010, and 2019. The ACI and RCI for ASR-prevalence (Supplemental Table 3) gradually decreased. Similarity or slow growth was observed in the ACI and RCI of ASR-death (Supplemental Table 4).

**Table 3:** Concentration indices for ASR-DALYs of maternal obstructed labor and uterine rupture in 1990 and 2019

	1990			2019		
	Estimate	95% CI lower	95% CI upper	Estimate	95% CI lower	95% CI upper
Difference	51.02	29.17	72.87	36.28	22.28	50.29
Ratio	42.93	19.21	95.94	37.38	17.30	80.77
Absolute concentration index	-10.25	-11.68	-8.82	-7.68	-8.39	-6.97
Relative concentration index	-59.15	-67.60	-50.71	-59.43	-65.75	-53.10
Population attributable fraction	-92.98	-93.02	-92.93	-92.28	-92.33	-92.23
Population attributable risk	-16.11	-16.11	-16.10	-11.93	-11.93	-11.92

ASR, age-standardized rate; DALYs, disability-adjusted life years; CI, confidence interval

## Discussion

Maternal OL and UR rates have dropped globally, yet the impact in terms of DALYs and mortality is significant. Health inequality studies highlight regional, age, population, and socio-economic factors influencing the disease burden, particularly in low SDI areas.

This study provided a comprehensive analysis of the burden and trends of OL and UR. The global burden of OL and UR has decreased significantly over the past few decades, as evidenced by a decline in incidence and prevalence, which may be related to a number of factors. First, advances in medical technology may have contributed to earlier identification and management of these problems [10, 11]. Second, more prenatal testing and pregnancy care may have helped reduce the chances of these problems occurring [6]. Further, the availability of new reproductive and medical technologies may also help reduce these problems [12]. However, despite these improvements, the burden of disease, as measured by the number of DALYs and deaths, remains significant. This suggests that despite the progress in reducing OL and UR incidence, the severity and impact of these complications on maternal health outcomes remains significant [13].

Age group analysis showed that women in their 20s and 30s are more likely to experience these obstetric complications. This may be due to several factors, including physiological changes during labor and delivery, lack of access to quality health care, and low awareness of the risks of childbirth [14, 15]. In addition, the study found an increase in the burden of DALYs in those aged 10–19 years old. This may be due to higher fertility rates among adolescents and young women, lack of proper birth knowledge, and ineffective antenatal checkups and care [8, 16]. Improvements in several areas such as education, policies, and services are needed to reduce the incidence of OL and UR and improve maternal health [13].

In terms of regional disparities, the burden and trends of OL and UR varied significantly across regions. Their incidence has increased in high-income countries in the Asia-Pacific region, Oceania, and Eastern and Western Europe, whereas DALYs have increased significantly in Andean Latin America, Caribbean, and Oceania. Possible reasons for these regional disparities include inadequate healthcare resources, such as inadequate obstetricians and facilities, low levels of women’s education, low incomes and lower socioeconomic status [17–20]. This suggests the need for further in-depth investigations and analyses to provide a more detailed and comprehensive understanding in order to develop more effective policies and interventions to ameliorate the differences in the burden of OL and UR across districts [21].

Many factors may contribute to the increased correlation of iron deficiency as a risk factor for OL and UR. Women who are less educated, have lower incomes or live in rural areas are more likely to experience iron deficiency [22]. It is important to strengthen maternal nutrition education and awareness, especially regarding iron intake and absorption [23]. Local health authorities should improve and strengthen health facilities, especially in resource-poor areas, to ensure that women have access to timely antenatal check-ups and medical services for early detection and management of problems related to iron deficiency.

The decomposition analysis provided insights into the factors driving the burden of OL and UR globally and in different levels of SDI. Population growth played a significant role in the burden of these conditions, particularly in low SDI regions [24]. This highlights the importance of addressing population growth as a potential strategy to reduce the burden of OL and UR in these regions [25]. Additionally, the analysis of health inequalities using slope indices and concentration indices provided valuable information on the disparities in disease burden across different SDI levels. The negative slope values indicated a reduction in disease burden with increasing SDI, suggesting an association between socio-economic development and improved maternal health outcomes [26]. The concentration indices showed a decrease in health inequality over time, indicating a gradual reduction in disparities in the burden of OL and UR across different socio-economic groups [24, 27]. These findings highlight the need for targeted interventions to address health inequalities and ensure equitable access to high-quality obstetric care.

There are several limitations that should be considered. First, the study relied on secondary data from the GBD database, which may be subject to reporting biases and data limitations. Second, the study focused on OL and UR as the main outcomes of interest, but there may be other contributing factors and complications that were not accounted for in the analysis. Finally, the study primarily focused on the burden and trends of OL and UR, as well as health inequalities associated with these conditions. Further research is required to explore the underlying factors and mechanisms that contribute to these outcomes, such as access to healthcare services, quality of care, and socio-economic determinants of health.

## Conclusions

This study provides a comprehensive analysis of the burden, risk factors, decomposition, and health inequalities associated with OL and UR. The incidence and prevalence have significantly decreased globally; however, the burden in terms of DALYs and deaths remains substantial. The analysis of health inequalities has revealed important information regarding regional disparities, aging, population growth, and socio-economic disparities that contribute to the burden of these conditions. Thees findings highlight the significant burden of these obstetric complications and emphasize the need for continued efforts to reduce maternal mortality and morbidity.

### Electronic supplementary material

Below is the link to the electronic supplementary material.

Supplementary Material 1

## Data Availability

All datasets used in this study are publicly available and can be accessed at https://vizhub.healthdata.org/gbd-results/. All code analyzed in R is available by contacting the corresponding author.

## Abbreviations

OL: Obstructed labor
UR: Uterine rupture
GBD: Global Burden of Disease
DALYs: Disability-adjusted life years
ASR: Age-standardized rates
SDI: Socio-demographic index
EAPC: Estimated annual percentage change
HEAT: Health Equity Assessment Toolkit
ACI: Absolute concentration index
RCI: Relative concentration index
PAF: Population attributable fraction
PAR: Population attributable risk
95%UI: 95% uncertainty interval
95%CI: 95% confidence interval
