# Supplementary material for: The global burden, risk and inequality of maternal obstructed labor and uterine rupture from 1990 to 2019
Source: BMC Public Health. 2024 Jul 29;24:2017. doi: 10.1186/s12889-024-19429-2 (PMC11285606; doi:10.1186/s12889-024-19429-2)
Supplement: Supplementary file 1 — Supplementary Material 1 [file 12889_2024_19429_MOESM1_ESM.docx]

# The Global Burden, Risk and Inequality of Maternal Obstructed Labor and Uterine Rupture from 1990 to 2019

## The list of files

**Supplemental Table 1** The number and ASR of prevalence and deaths for maternal obstructed labor and uterine rupture in 2019 and changing trends from 1990 to 2019

**Supplemental Table 2** Concentration Indices for ASIR of maternal obstructed labor and uterine rupture from 1990 to 2019

**Supplemental Table 3** Concentration Indices for ASPR of maternal obstructed labor and uterine rupture from 1990 to 2019

**Supplemental Table 4** Concentration Indices for ASDR of maternal obstructed labor and uterine rupture from 1990 to 2019

**Supplemental Figure 1** Line chart depicting global burden of maternal obstructed labor and uterine rupture incidence across different age groups from 1990 to 2019. (A) Number of incidences; (B) Incidence rate.

**Supplemental Figure 2** Line chart depicting global burden of maternal obstructed labor and uterine rupture DALYs across different age groups from 1990 to 2019. (A) Number of DALYs; (B) DALYs rate.

**Supplemental Figure 3** Line chart depicting global burden of maternal obstructed labor and uterine rupture prevalence across different age groups from 1990 to 2019. (A) Number of prevalence; (B) prevalence rate.

**Supplemental Figure 4** Line chart depicting global burden of maternal obstructed labor and uterine rupture deaths across different age groups from 1990 to 2019. (A) Number of deaths; (B) deaths rate.

**Supplemental Figure 5** Global distribution maps for the burden of maternal obstructed labor and uterine rupture in 204 countries and territories in 1990 and 2019. (A) ASR- prevalence in 1990; (B) ASR- prevalence in 2019; (C) ASR- deaths in 1990; (D) ASR- deaths in 2019. ASR, age-standardized rates.

**Supplemental Figure 6** Global distribution maps for the burden of maternal obstructed labor and uterine rupture in 204 countries and territories in 1990 and 2019. (A) number-incidence in 1990; (B) number-incidence in 2019; (C) number-DALYs in 1990; (D) number-DALYs in 2019. DALYs, disability-adjusted life years.

**Supplemental Figure 7** Global distribution maps for the burden of maternal obstructed labor and uterine rupture in 204 countries and territories in 1990 and 2019. (A) number- prevalence in 1990; (B) number-prevalence in 2019; (C) number-deaths in 1990; (D) number-deaths in 2019.

**Supplemental Figure 8** Changes in maternal obstructed labor and uterine rupture DALYs rate according to population-level determinants of population growth, aging, and epidemiological change from 1990 to 2019 at the global Level and by SDI quintile. (The black dot represents the overall value of change contributed by all 3 components.) DALYs, disability-adjusted life years; SDI, Sociodemographic Index.

**Supplemental Figure 9** Changes in maternal obstructed labor and uterine rupture prevalence rate according to population-level determinants of population growth, aging, and epidemiological change from 1990 to 2019 at the global Level and by SDI quintile. (The black dot represents the overall value of change contributed by all 3 components.) SDI, Sociodemographic Index.

**Supplemental Figure 10** Changes in maternal obstructed labor and uterine rupture deaths rate according to population-level determinants of population growth, aging, and epidemiological change from 1990 to 2019 at the global Level and by SDI quintile. (The black dot represents the overall value of change contributed by all 3 components.) SDI, Sociodemographic Inde

**Supplemental table 1 The number and ASR of prevalence and deaths for maternal obstructed labor and uterine rupture in 2019 and changing trends from 1990 to 2019**

|  | 2019 | | 1990-2019 | 2019 | | 1990-2019 |
| --- | --- | --- | --- | --- | --- | --- |
|  | prevalence cases | ASPR per 100,000 (95% UI) | EAPC of ASPR (95% CI) | Deaths cases | ASDR per 100,000 (95% UI) | EAPC of ASDR (95% CI) |
| Global | 1,235,134.96 (1,577,169.65 - 940,061.37) | 15.96 (20.38 - 12.15) | -1.55 (-1.68 to -1.42) | 10,534.64 (12,509.10 - 8,831.68) | 0.14 (0.16 - 0.11) | -0.37 (-0.9 to 0.15) |
| Age (years) |  |  |  |  |  |  |
| 0-9 years | 0.00 (0.00 - 0.00) | 0.00 (0.00 - 0.00) | 0.00 (0.00 - 0.00) | 0.00 (0.00 - 0.00) | 0.00 (0.00 - 0.00) | 0.00 (0.00 - 0.00) |
| 10-14 years | 803.57 (1,390.66 - 433.26) | 0.13 (0.22 - 0.07) | -0.1 (-0.24 to 0.04) | 33.70 (47.77 - 24.01) | 0.01 (0.01 - 0.00) | 2.34 (1.8 to 2.88) |
| 15-19 years | 44,029.43 (66,745.76 - 26,553.55) | 7.11 (10.77 - 4.29) | -0.9 (-1.01 to -0.79) | 825.54 (1,054.83 - 643.00) | 0.13 (0.17 - 0.10) | 0.37 (-0.34 to 1.09) |
| 20-24 years | 147,085.01 (206,150.89 - 96,895.71) | 24.51 (34.35 - 16.15) | -1.12 (-1.2 to -1.05) | 1,811.27 (2,378.07 - 1,361.32) | 0.30 (0.40 - 0.23) | -0.98 (-1.71 to -0.25) |
| 25-29 years | 224,017.75 (298,713.94 - 156,850.50) | 37.00 (49.34 - 25.91) | -1.36 (-1.44 to -1.29) | 1,948.45 (2,493.34 - 1,519.93) | 0.32 (0.41 - 0.25) | -1.1 (-1.68 to -0.52) |
| 30-34 years | 276,056.38 (393,187.49 - 186,666.44) | 45.88 (65.34 - 31.02) | -1.49 (-1.69 to -1.29) | 1,998.24 (2,535.46 - 1,574.44) | 0.33 (0.42 - 0.26) | -0.62 (-1.18 to -0.06) |
| 35-39 years | 221,960.50 (311,132.57 - 154,094.12) | 41.03 (57.51 - 28.48) | -1.49 (-1.72 to -1.25) | 1,851.55 (2,347.11 - 1,467.18) | 0.34 (0.43 - 0.27) | 0.15 (-0.42 to 0.72) |
| 40-44 years | 141,595.35 (193,762.95 - 101,876.07) | 28.70 (39.27 - 20.65) | -1.64 (-1.85 to -1.42) | 1,315.47 (1,658.44 - 1,050.00) | 0.27 (0.34 - 0.21) | 0.28 (-0.14 to 0.7) |
| 45-49 years | 81,572.90 (110,323.69 - 58,361.86) | 17.22 (23.28 - 12.32) | -2.08 (-2.3 to -1.85) | 713.35 (948.81 - 543.19) | 0.15 (0.20 - 0.11) | 0.71 (0.37 to 1.06) |
| 50-54 years | 41,586.54 (56,726.78 - 30,685.73) | 9.52 (12.99 - 7.02) | -2.44 (-2.63 to -2.26) | 37.08 (48.68 - 28.57) | 0.01 (0.01 - 0.01) | 0.92 (0.33 to 1.51) |
| 55+ years | 0.00 (0.00 - 0.00) | 0.00 (0.00 - 0.00) | 0.00 (0.00 - 0.00) | 0.00 (0.00 - 0.00) | 0.00 (0.00 - 0.00) | 0.00 (0.00 - 0.00) |
| SDI |  |  |  |  |  |  |
| High | 16,824.56 (25,483.46 - 9,894.29) | 1.66 (2.51 - 0.98) | -1.51 (-1.75 to -1.27) | 21.38 (26.25- 17.47) | 0.00 (0.00 - 0.00) | -0.93 (-1.15 to -0.71) |
| High-middle | 35,165.35 (46,220.21 - 25,501.30) | 2.46 (3.23 - 1.78) | -0.95 (-1.09 to -0.81) | 268.84 (347.50 - 207.24) | 0.02 (0.02 - 0.01) | -3.65 (-4 to -3.3) |
| Middle | 165,209.31 (210,298.56 - 125,654.12) | 6.89 (8.77 - 5.24) | -1.02 (-1.17 to -0.86) | 1,581.13 (1,956.75 - 1,255.46) | 0.07 (0.08 - 0.05) | -2.13 (-2.33 to -1.94) |
| Low-middle | 466,621.01 (614,415.32 - 347,893.68) | 26.45 (34.83 - 19.72) | -2.98 (-3.14 to -2.81) | 4,133.37 (4,993.29 - 3,337.86) | 0.23 (0.28 - 0.19) | -0.97 (-1.76 to -0.19) |
| Low | 550,944.11 (697,212.34 - 419,585.19) | 48.81 (61.77 - 37.17) | -2.25 (-2.3 to -2.21) | 4,523.03 (5,480.92 - 3,716.61) | 0.40 (0.49 - 0.33) | -0.13 (-0.58 to 0.33) |
| GBD region |  |  |  |  |  |  |
| Central Asia | 1,793.64 (2,817.69 - 1,043.33) | 1.92 (3.01 - 1.12) | -0.71 (-1.03 to -0.4) | 7.20 (9.38 - 5.68) | 0.01 (0.01 - 0.01) | -1.56 (-2.17 to -0.95) |
| East Asia | 11,112.82 (17,118.45 - 6,575.73) | 0.75 (1.16 - 0.45) | -1.74 (-2.12 to -1.36) | 133.83 (171.18 - 101.78) | 0.01 (0.01 - 0.01) | -4.82 (-5.57 to -4.07) |
| South Asia | 518,977.27 (696,315.82 - 380,856.10) | 28.75 (38.57 - 21.10) | -3.5 (-3.64 to -3.37) | 3,587.64 (4,695.86 - 2,714.67) | 0.20 (0.26 - 0.15) | -3.42 (-4.56 to -2.27) |
| Southeast Asia | 9,196.53 (14,588.02 - 5,342.26) | 1.36 (2.17 - 0.79) | -1.55 (-1.6 to -1.49) | 192.94 (239.64 - 154.80) | 0.03 (0.04 - 0.02) | -1.51 (-2.04 to -0.97) |
| High-income Asia Pacific | 1,437.71 (2,274.77 - 838.89) | 0.77 (1.21 - 0.45) | 0.18 (-0.1 to 0.47) | 1.54 (1.99 - 1.18) | 0.00 (0.00 - 0.00) | -5.55 (-5.88 to -5.21) |
| North Africa and Middle East | 71,592.79 (89,970.62 -55,468.15) | 11.76 (14.78 - 9.11) | -3.53 (-3.58 to -3.48) | 696.21 (906.22 - 535.83) | 0.11 (0.15 - 0.09) | -0.43 (-0.52 to -0.35) |
| Central Sub-Saharan Africa | 29,838.44 (40,407.78 - 21,649.66) | 22.68 (30.72 - 16.46) | -2.98 (-3.27 to -2.7) | 205.05 (274.93 - 144.42) | 0.16 (0.21 - 0.11) | 1.78 (1.19 to 2.37) |
| Eastern Sub-Saharan Africa | 299,060.41 (384,752.52 - 224,085.70) | 72.63 (93.44 - 54.42) | -1.8 (-1.87 to -1.74) | 2,533.11 (3,121.28 - 1,953.86) | 0.62 (0.76 - 0.47) | 1 (0.55 to 1.45) |
| Southern Sub-Saharan Africa | 25,666.83 (34,634.82 - 18,446.50) | 32.67 (44.08 - 23.48) | -1.65 (-1.91 to -1.39) | 78.02 (104.01 - 57.20) | 0.10 (0.13 - 0.07) | -0.32 (-1.45 to 0.82) |
| Western Sub-Saharan Africa | 233,357.03 (291.918.47 - 178,060.66) | 51.14 (63.97 - 39.02) | -0.41 (-0.5 to -0.32) | 2,829.91 (3,834.66 - 2,089.42) | 0.62 (0.84 - 0.46) | -0.01 (-0.19 to 0.18) |
| Andean Latin America | 1,796.83 (2,744.98 - 1,044.18) | 2.83 (4.32 - 1.64) | -1.59 (-1.7 to -1.49) | 23.53 (31.31 - 16.99) | 0.04 (0.05 - 0.03) | 12.02 (10.07 to 14) |
| Central Latin America | 4,258.10 (6,447.94 - 2,573.95) | 1.70 (2.58 - 1.03) | -2.05 (-2.25 to -1.86) | 96.39 (120.61 - 75.56) | 0.04 (0.05 - 0.03) | -1 (-1.22 to -0.78) |
| Southern Latin America | 3,315.54 (4,928.01 - 1,997.25) | 4.97 (7.38 - 2.99) | -0.91 (-1.02 to -0.81) | 4.34 (5.58 - 3.38) | 0.01 (0.01 - 0.01) | 7.4 (5.85 to 8.96) |
| Tropical Latin America | 1,271.97 (1898.86 - 764.46) | 0.57 (0.85 - 0.34) | -1.22 (-1.4 to -1.04) | 39.51 (50.35 - 30.11) | 0.02 (0.02 - 0.01) | -2.57 (-2.91 to -2.22) |
| High-income North America | 6,692.52 (10,099.23 - 3,976.27) | 1.84 (2.77 - 1.09) | -3 (-3.55 to -2.44) | 8.34 (11.02 - 6.26) | 0.00 (0.00 - 0.00) | 4.3 (3.27 to 5.34) |
| Caribbean | 550.56 (860.65 - 320.35) | 1.17 (1.82 - 0.68) | -1.04 (-1.1 to -0.97) | 11.78 (15.60 - 8.99) | 0.02 (0.03 - 0.02) | 8.38 (6.87 to 9.91) |
| Australasia | 1,009.68 (1,542.43 - 573.82) | 3.47 (5.31 - 1.97) | 0.29 (0.16 to 0.42) | 0.12 (0.16 - 0.09) | 0.00 (0.00 - 0.00) | -1.17 (-2.09 to -0.23) |
| Oceania | 342.51 (544.69 - 203.77) | 2.58 (4.10 - 1.53) | -0.43 (-0.45 to -0.41) | 73.34 (105.95 - 49.41) | 0.55 (0.80 - 0.37) | 0.23 (-0.06 to 0.52) |
| Central Europe | 585.29 (919.97 - 337.94) | 0.51 (0.81 - 0.30) | -0.56 (-0.95 to -0.18) | 1.51 (1.82 - 1.23) | 0.00 (0.00 - 0.00) | -1.12 (-1.52 to -0.73) |
| Eastern Europe | 3,662.24 (5,777.45 - 2,075.35) | 1.74 (2.75 - 0.99) | 0.44 (-0.09 to 0.98) | 7.99 (10.83 - 5.71) | 0.00 (0.01 - 0.00) | -4.27 (-4.84 to -3.7) |
| Western Europe | 9,616.24 (14,648.59 - 5,491.63) | 2.20 (3.36 - 1.26) | 0.37 (0.25 to 0.49) | 2.35 (2.65 - 2.06) | 0.00 (0.00 - 0.00) | -3.23 (-3.57 to -2.89) |

ASR, age-standardized rate; ASPR, age-standardized prevalence rate; ASDR, age-standardized deaths rate; EAPC, estimated annual percentage change; UI, uncertainty interval; CI, confidence interval; SDI, Sociodemographic Index; GBD, global burden of disease.

**Supplemental table 2 Concentration Indices for ASIR of maternal obstructed labor and uterine rupture from 1990 to 2019**

|  | Difference | Ratio | Absolute concentration index | Relative concentration index |
| --- | --- | --- | --- | --- |
| 1990 |  |  |  |  |
| estimate | -34.92 | 0.88 | -17.25 | -8.62 |
| 95% CI lower bound | -118.11 | 0.65 | -24.42 | -9.28 |
| 95% CI upper bound | 48.27 | 1.19 | -10.09 | -7.96 |
| 2000 |  |  |  |  |
| estimate | -23.19 | 0.92 | -14.51 | -8.97 |
| 95% CI lower bound | -83.39 | 0.73 | -20.31 | -9.60 |
| 95% CI upper bound | 37.02 | 1.16 | -8.71 | -8.35 |
| 2010 |  |  |  |  |
| estimate | -50.09 | 0.82 | -15.33 | -10.93 |
| 95% CI lower bound | -104.04 | 0.66 | -19.67 | -11.63 |
| 95% CI upper bound | 3.87 | 1.03 | -10.99 | -10.23 |
| 2019 |  |  |  |  |
| estimate | -10.09 | 0.96 | -13.22 | -10.86 |
| 95% CI lower bound | -78.95 | 0.71 | -16.68 | -11.54 |
| 95% CI upper bound | 58.77 | 1.29 | -9.75 | -10.19 |

ASIR, age-standardized incidences rate; CI, confidence interval.

**Supplemental table 3 Concentration Indices for ASPR of maternal obstructed labor and uterine rupture from 1990 to 2019**

|  | Difference | Ratio | Absolute concentration index | Relative concentration index |
| --- | --- | --- | --- | --- |
| 1990 |  |  |  |  |
| estimate | 129.52 | 33.95 | -15.71 | -61.19 |
| 95% CI lower bound | 87.63 | 19.47 | -18.01 | -70.73 |
| 95% CI upper bound | 171.41 | 59.20 | -13.40 | -51.65 |
| 2000 |  |  |  |  |
| estimate | 112.70 | 30.16 | -12.21 | -59.11 |
| 95% CI lower bound | 75.85 | 18.07 | -13.77 | -67.56 |
| 95% CI upper bound | 149.55 | 50.34 | -10.65 | -50.66 |
| 2010 |  |  |  |  |
| estimate | 97.60 | 25.79 | -10.66 | -57.12 |
| 95% CI lower bound | 65.15 | 15.61 | -11.86 | -64.76 |
| 95% CI upper bound | 130.05 | 42.60 | -9.45 | -49.47 |
| 2019 |  |  |  |  |
| estimate | 84.42 | 27.29 | -8.82 | -55.20 |
| 95% CI lower bound | 54.77 | 15.48 | -9.71 | -62.21 |
| 95% CI upper bound | 114.07 | 48.12 | -7.93 | -48.20 |

ASPR, age-standardized prevalence rate; CI, confidence interval.

**Supplemental table 4 Concentration Indices for ASDR of maternal obstructed labor and uterine rupture from 1990 to 2019**

|  | Difference | Ratio | Absolute concentration index | Relative concentration index |
| --- | --- | --- | --- | --- |
| 1990 |  |  |  |  |
| estimate | 0.18 | 492.13 | -0.09 | -57.43 |
| 95% CI lower bound | 0.09 | 270.89 | -0.10 | -65.52 |
| 95% CI upper bound | 0.27 | 894.05 | -0.08 | -49.34 |
| 2000 |  |  |  |  |
| estimate | 0.12 | 270.01 | -0.06 | -53.91 |
| 95% CI lower bound | 0.06 | 150.92 | -0.07 | -60.25 |
| 95% CI upper bound | 0.18 | 483.07 | -0.06 | -47.56 |
| 2010 |  |  |  |  |
| estimate | 0.15 | 398.41 | -0.07 | -60.48 |
| 95% CI lower bound | 0.07 | 221.69 | -0.08 | -67.34 |
| 95% CI upper bound | 0.22 | 716.01 | -0.06 | -53.61 |
| 2019 |  |  |  |  |
| estimate | 0.16 | 539.71 | -0.08 | -62.38 |
| 95% CI lower bound | 0.09 | 307.98 | -0.09 | -69.99 |
| 95% CI upper bound | 0.24 | 945.79 | -0.08 | -54.77 |

ASDR, age-standardized deaths rate; CI, confidence interval.


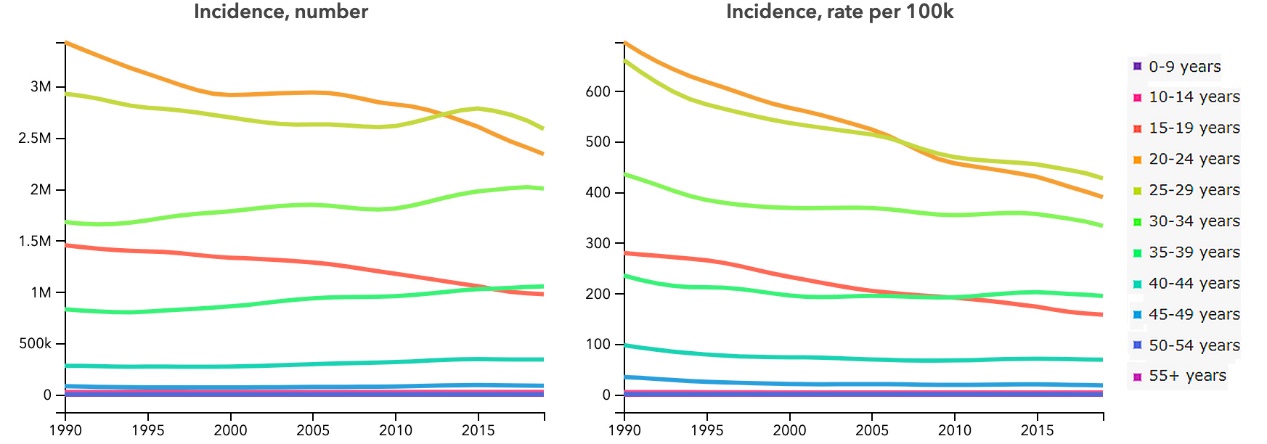


**Supplemental Figure 1** Line chart depicting global burden of maternal obstructed labor and uterine rupture incidence across different age groups from 1990 to 2019.

(A) Number of incidences; (B) Incidence rate.

**
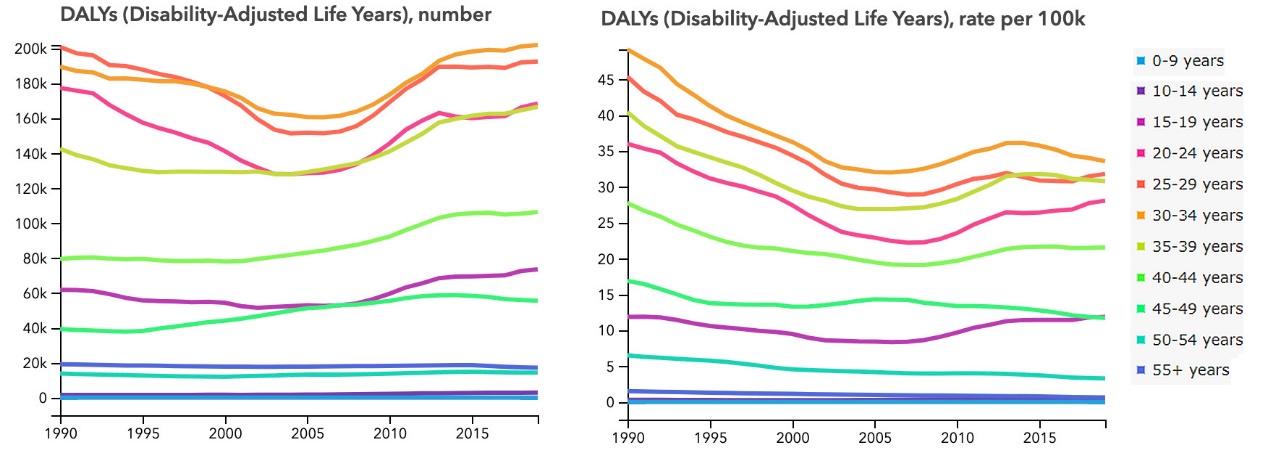
**

**Supplemental Figure 2** Line chart depicting global burden of maternal obstructed labor and uterine rupture DALYs across different age groups from 1990 to 2019.

(A) Number of DALYs; (B) DALYs rate.


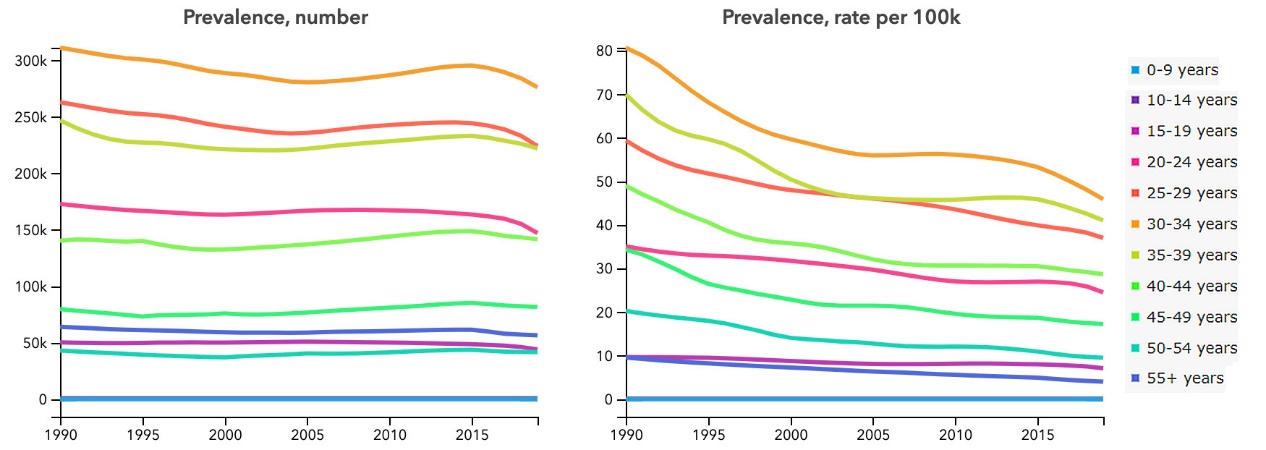


**Supplemental Figure 3** Line chart depicting global burden of maternal obstructed labor and uterine rupture prevalence across different age groups from 1990 to 2019.

(A) Number of prevalence; (B) prevalence rate.


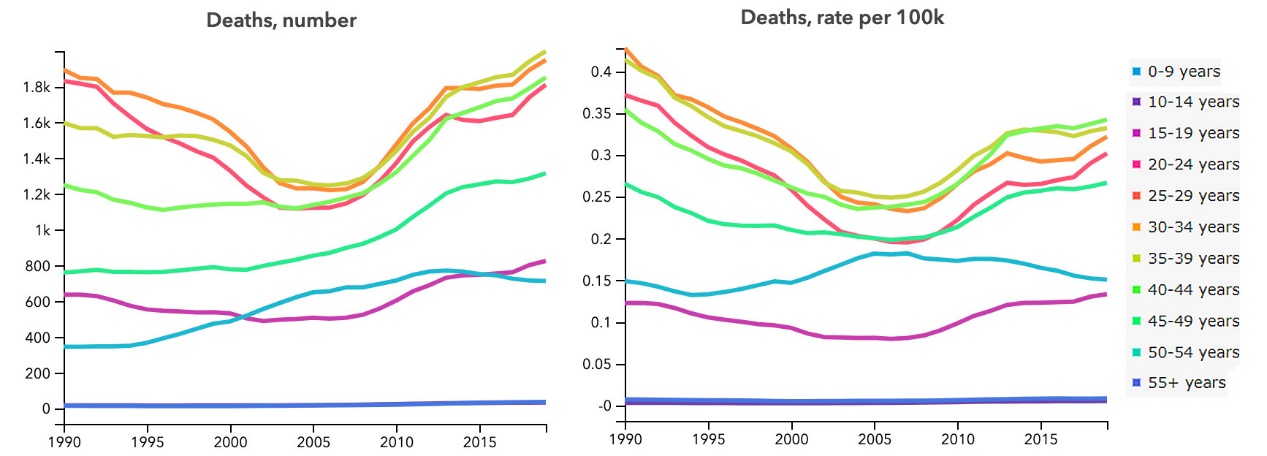


**Supplemental Figure 4** Line chart depicting global burden of maternal obstructed labor and uterine rupture deaths across different age groups from 1990 to 2019.

(A) Number of deaths; (B) deaths rate.


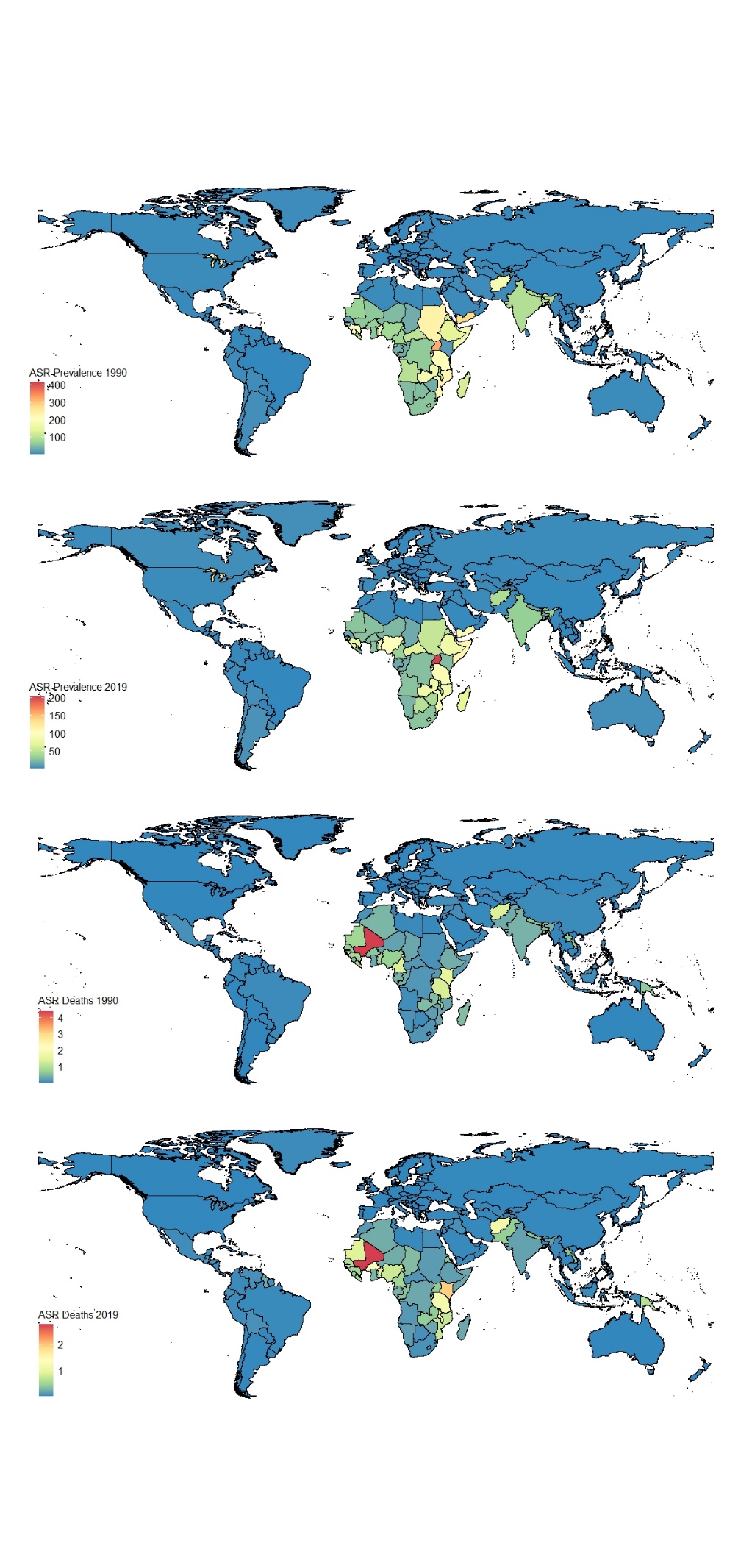


**Supplemental Figure 5** Global distribution maps for the burden of maternal obstructed labor and uterine rupture in 204 countries and territories in 1990 and 2019.

(A) ASR- prevalence in 1990; (B) ASR- prevalence in 2019; (C) ASR- deaths in 1990; (D) ASR- deaths in 2019. ASR, age-standardized rates.


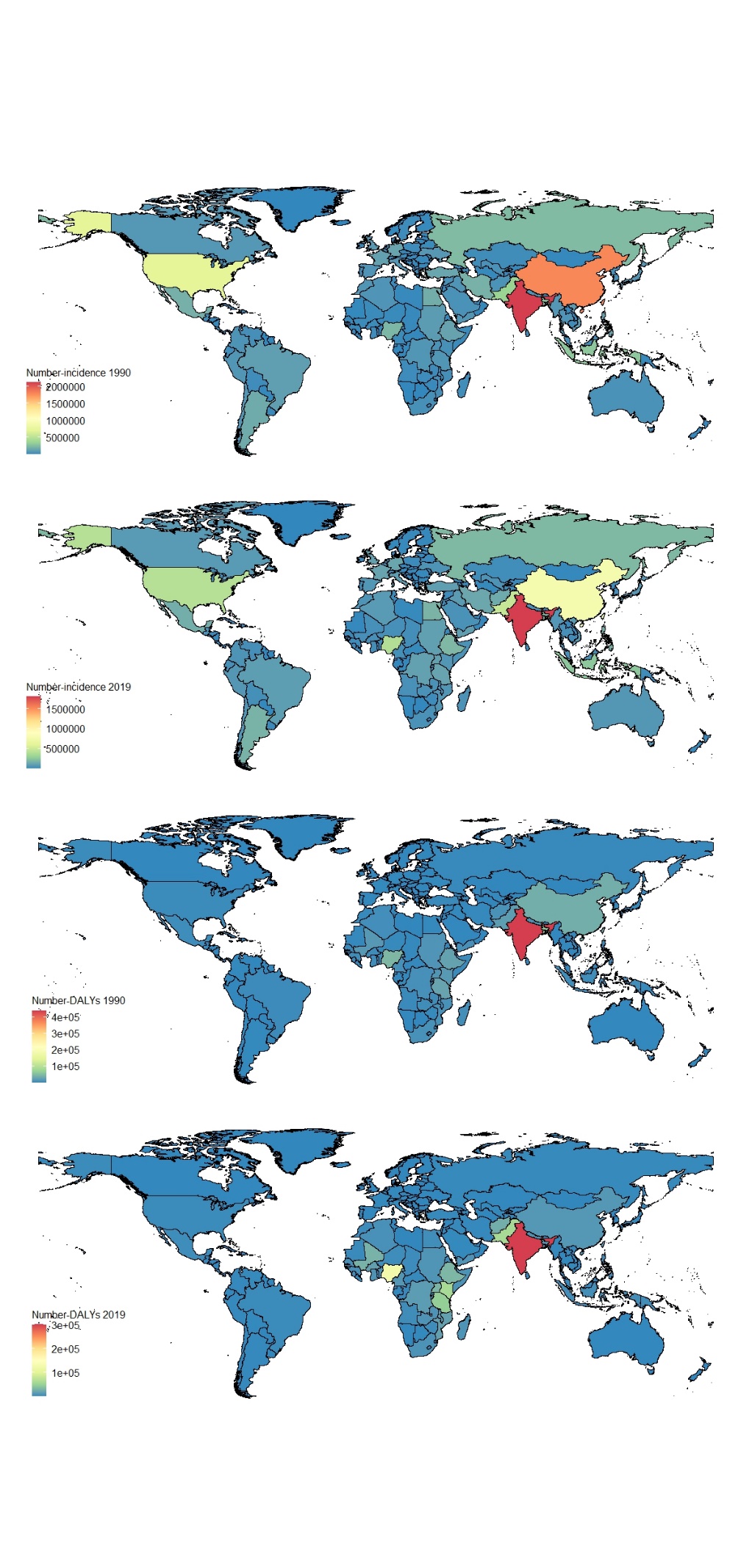


**Supplemental Figure 6** Global distribution maps for the burden of maternal obstructed labor and uterine rupture in 204 countries and territories in 1990 and 2019.

(A) number-incidence in 1990; (B) number-incidence in 2019; (C) number-DALYs in 1990; (D) number-DALYs in 2019. DALYs, disability-adjusted life years.


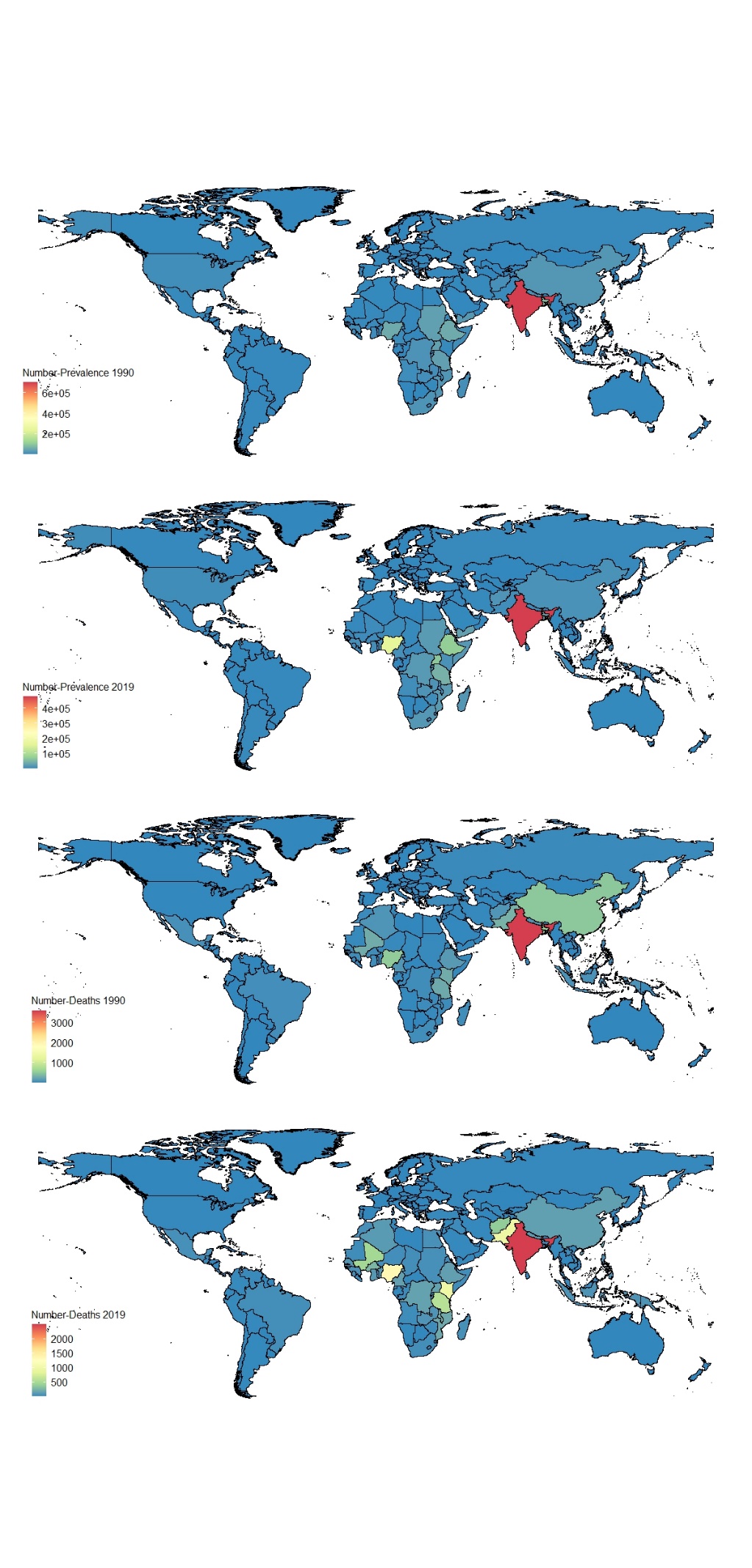


**Supplemental Figure 7** Global distribution maps for the burden of maternal obstructed labor and uterine rupture in 204 countries and territories in 1990 and 2019.

(A) number- prevalence in 1990; (B) number-prevalence in 2019; (C) number-deaths in 1990; (D) number-deaths in 2019.


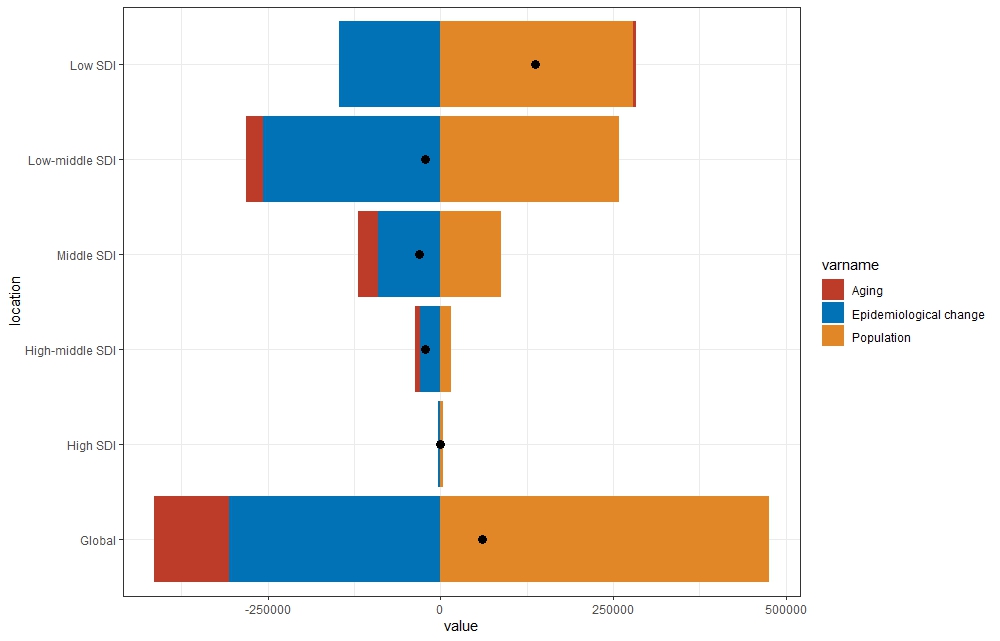


**Supplemental Figure 8** Changes in maternal obstructed labor and uterine rupture DALYs rate according to population-level determinants of population growth, aging, and epidemiological change from 1990 to 2019 at the global Level and by SDI quintile. (The black dot represents the overall value of change contributed by all 3 components.) DALYs, disability-adjusted life years; SDI, Sociodemographic Index.


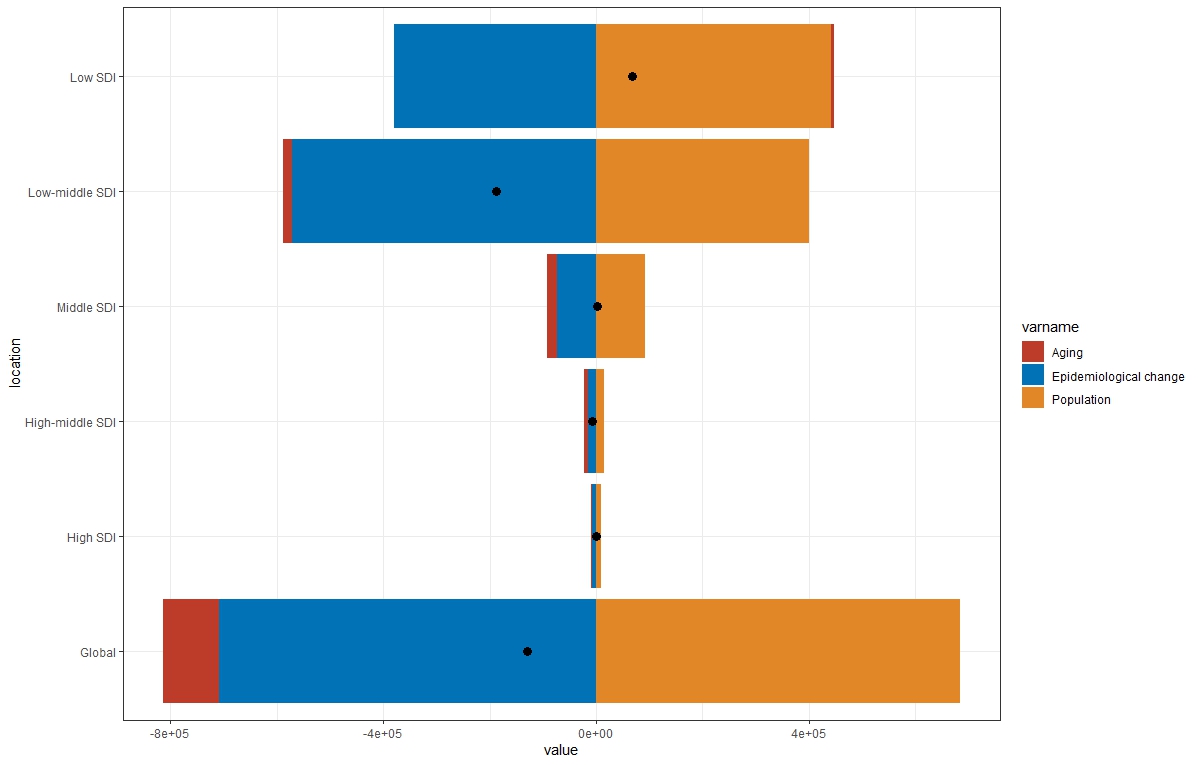


**Supplemental Figure 9** Changes in maternal obstructed labor and uterine rupture prevalence rate according to population-level determinants of population growth, aging, and epidemiological change from 1990 to 2019 at the global Level and by SDI quintile. (The black dot represents the overall value of change contributed by all 3 components.) SDI, Sociodemographic Index.


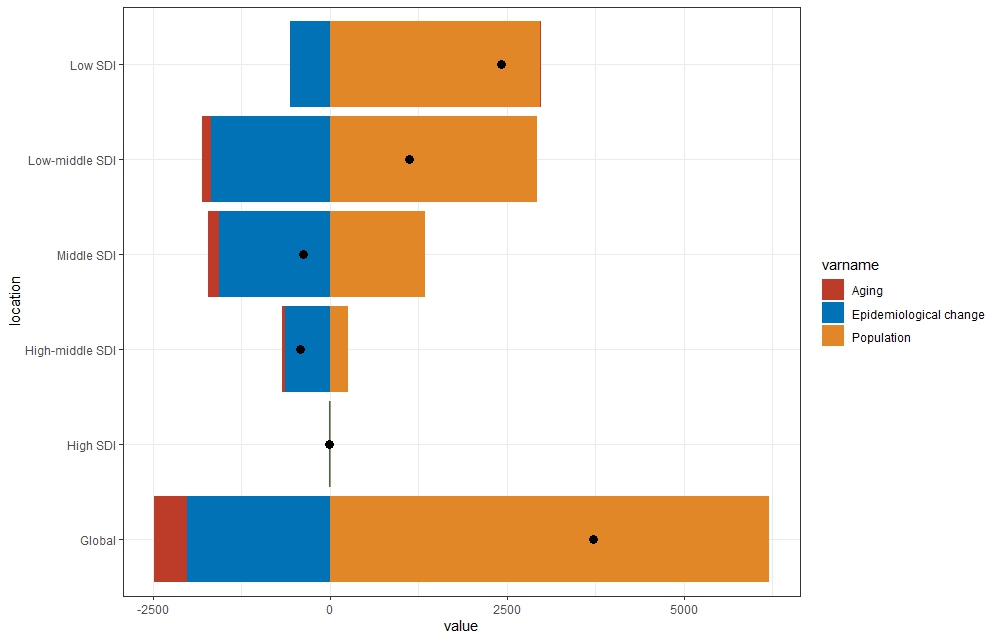


**Supplemental Figure 10** Changes in maternal obstructed labor and uterine rupture deaths rate according to population-level determinants of population growth, aging, and epidemiological change from 1990 to 2019 at the global Level and by SDI quintile. (The black dot represents the overall value of change contributed by all 3 components.) SDI, Sociodemographic Index.
